# Description of *Parvocalanus leei* sp. n. (Copepoda, Calanoida, Paracalanidae) in Western Korea, with comments on the taxonomic position of *Paracalanus arabiensis* Kesarkar & Anil, 2010

**DOI:** 10.3897/zookeys.456.7741

**Published:** 2014-11-21

**Authors:** Seong Yong Moon, Seok-Hyun Youn, Ho Young Soh

**Affiliations:** 1 Fisheries and Ocean Information Division, National Fisheries Research and Development Institute, Busan 619-705, South Korea; 2 Faculty of Marine Technology, Chonnam National University, Yeosu 550-749, South Korea

**Keywords:** Copepoda, Calanoida, Paracalanidae, *Parvocalanus*, *Paracalanus*, new species, taxonomy, Korea

## Abstract

A new species of paracalanid calanoid copepod *Parvocalanus leei* **sp. n.**, is described from specimens collected in shallow waters of Western Korea. The new species is closely similar to *Parvocalanus arabiensis* (Kesarkar & Anil, 2010), *Parvocalanus crassirostris* (F. Dahl, 1894), *Parvocalanus latus* Andronov, 1972, and *Parvocalanus scotti* (Früchtl, 1923) in having two short terminal spines on the distal segment of the fifth leg and a similar rostrum in the female, but can be readily distinguished from its congeners by the body size, relative length of antennules, segmentation of endopod of leg 1, and pattern of ornamentation of spinules on legs 1 to 4 in the female. The taxonomic position of *Parvocalanus arabiensis* and the validity of the genus *Parvocalanus* Andronov, 1970 are also discussed.

## Introduction

Most of the species of the family Paracalanidae Giesbrecht, 1892 are distributed across both northern and southern hemispheres, with about 80 species in eight genera having been described thus far (Boxshall and Halsey 2004; Vives and Shmeleva 2007; Bradford-Grieve 2008). Six out of the eight paracalanid genera accounting 12 species have been recorded thus far in Korean waters (*Acrocalanus* Giesbrecht, 1888, *Bestiolina* Andronov, 1991, *Calocalanus* Giesbrecht, 1888, *Mecynocera* I. C. Thompson, 1888, *Paracalanus* Boeck, 1865, and *Parvocalanus* Andronov, 1970) (Yoo 1995; Soh et al. 2013). The genus *Parvocalanus* Andronov, 1970 contains six valid species (Boxshall and Halsey 2004). It is one of the major constituents of the calanoid assemblage of shallow waters worldwide (Robertson et al. 1988; Beltrão et al. 2011; Chen et al. 2011). They are generally small-sized, varying from 0.5 to 1.5 mm in body length, and play an important role as primary consumers in marine ecosystems (Araujo 2006; Lenz 2012). In Korean waters, two species of *Parvocalanus* have been reported thus far: *Parvocalanus crassirostris* (F. Dahl, 1894) (as *Paracalanus crassirostris*) (Yoo, 1995) and *Parvocalanus elegans* Andronov, 1972 (Soh et al. 2013). They share many of the characteristics of *Paracalanus* Boeck, 1865, but differ in the following features: a short, blunt rostrum; short terminal spines on the distal segment of female P5; and absence of dorsal hump in the male (Andronov 1970). These prominent features were used by Andronov (1970) to transfer *Paracalanus crassirostris*, *Paracalanus dubia*, *Paracalanus scotti*, and *Paracalanus serratipes* to the genus *Parvocalanus*. However, most of *Parvocalanus* species are difficult to identify because of their similar aspect and poor original descriptions.

During an investigation of planktonic copepods collected in shallow waters of Western Korea, we identified a new species of *Parvocalanus* which had been previously overlooked. In this study, we describe it and provide keys to all genera of Paracalanidae and species within *Parvocalanus*. Additionally, we evaluate the taxonomic position of *Paracalanus arabiensis* Kesarkar & Anil, 2010.

## Materials and methods

**Figure 1.**
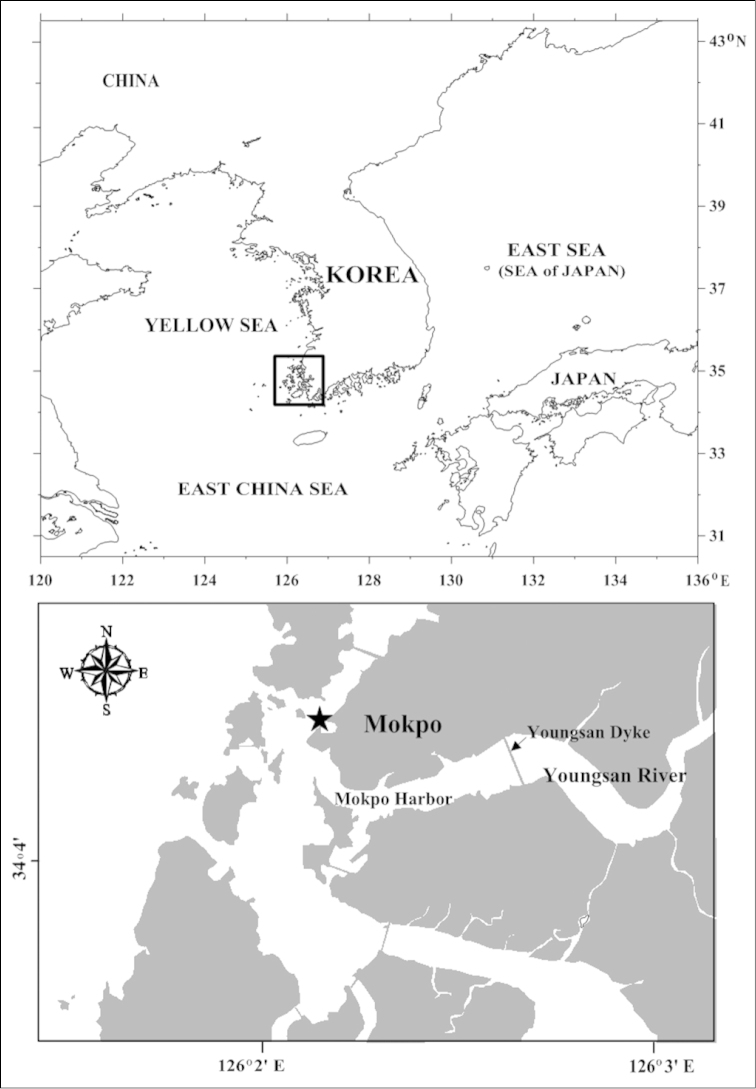
Map of sampling stations in the shallow waters of Mokpo, southern Korea.

Copepods were collected in shallow waters at Mokpo, Western Korea (Fig. 1), on 23 August 2013 with a 0.2 mm mesh-size plankton net. For morphological examination, samples were fixed in 5% natural formalin-seawater solution, and later cleared in 70% lactic acid for 1 to 2 hours before dissection in a drop of lactophenol using a wooden slide procedure (Humes and Gooding 1964). Dissected body parts and appendages were examined under a compound microscope up to × 1,000. Drawings were made with the aid of a drawing tube equipped on the microscope. Body size of individuals was measured from the head to tip of the caudal rami excluding caudal setae using a stage micrometer. Morphological terminology followed Huys and Boxshall (1991). Abbreviations used in text and figures are as follows: s, seta; ae, aesthetasc; P1–P5, legs 1 to 5, respectively. Specimens are deposited in the National Institute of Biological Resources (NIBR), Incheon, Korea.

## Systematics

### Order Calanoida G. O. Sars, 1903 Family Paracalanidae Giesbrecht, 1893 Genus *Parvocalanus* Andronov, 1970

#### Parvocalanus leei sp. n.

Taxon classificationAnimaliaCalanoidaParacalanidae

http://zoobank.org/C45C228A-4E84-4E83-95F1-D2FAEE57F0D0

**Figure 2.**
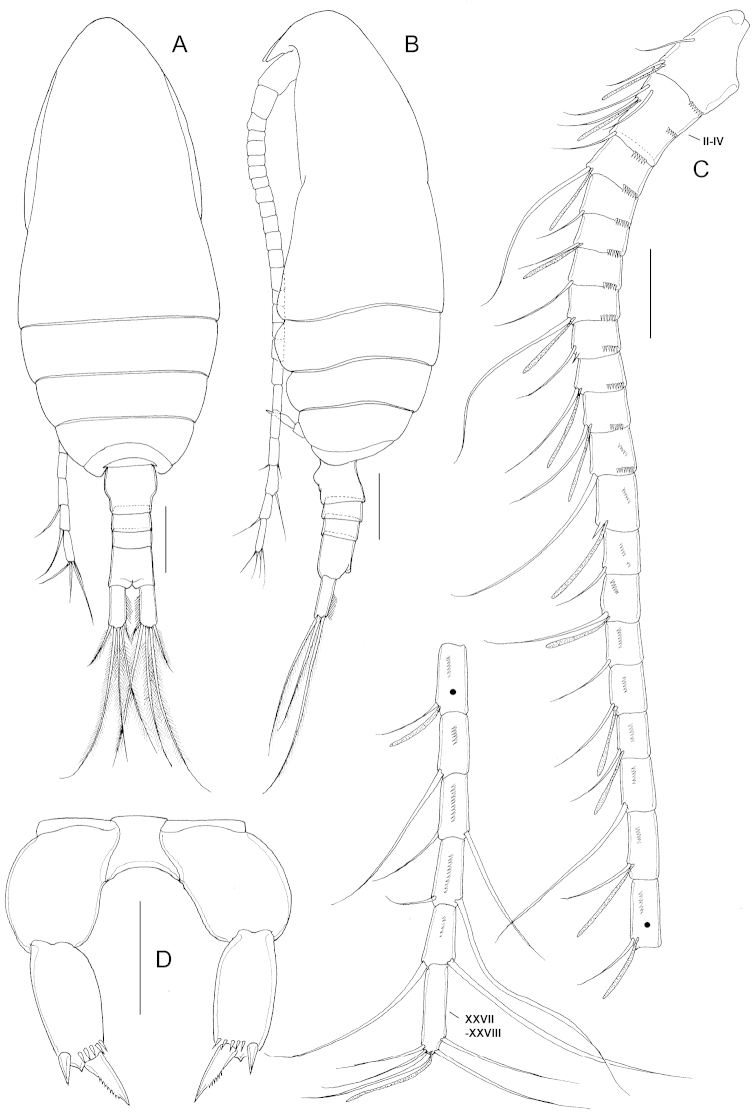
*Parvocalanus leei* sp. n., paratype adult female. **A** habitus, dorsal view **B** habitus, lateral view **C** Antennule **D** P5. Scale bars: **A, B** = 0.1 mm; **C** = 0.05 mm; **D** = 0.025 mm.

**Figure 3.**
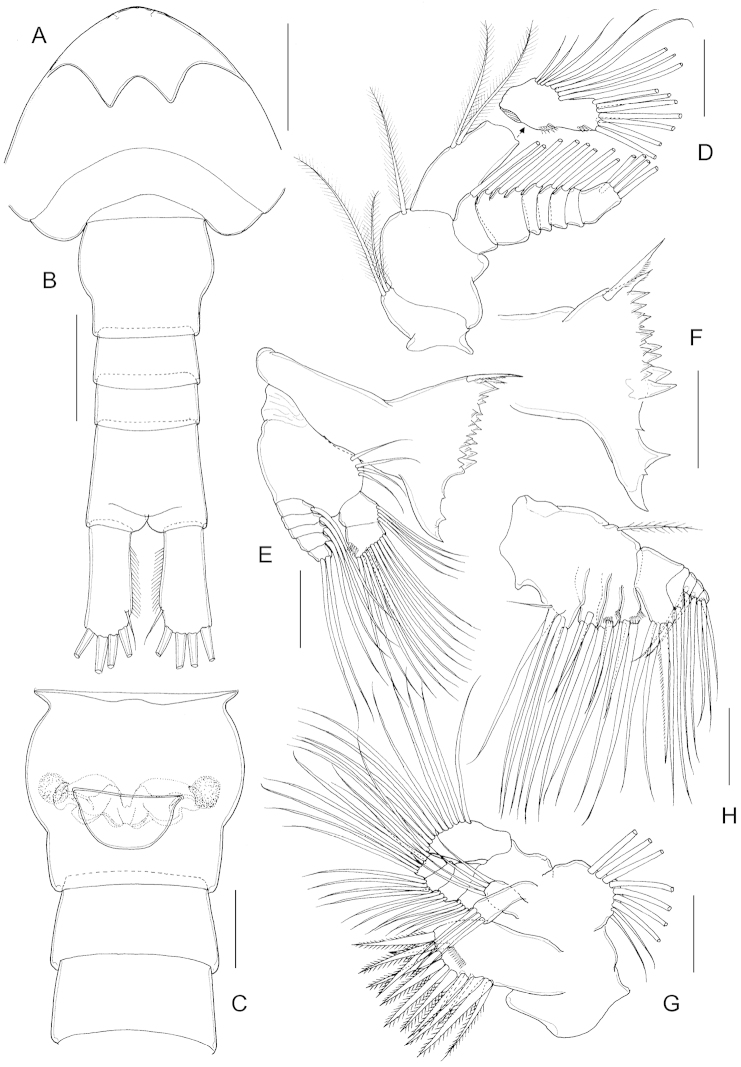
*Parvocalanus leei* sp. n., paratype adult female. **A** rostrum, ventral view **B** urosome, dorsal view **C** genital double-somite, ventral view **D** antenna **E** mandible **F** mandibular palp **G** maxillule. Scale bars: **A–C** = 0.05 mm; **D–G** = 0.025 mm.

**Figure 4.**
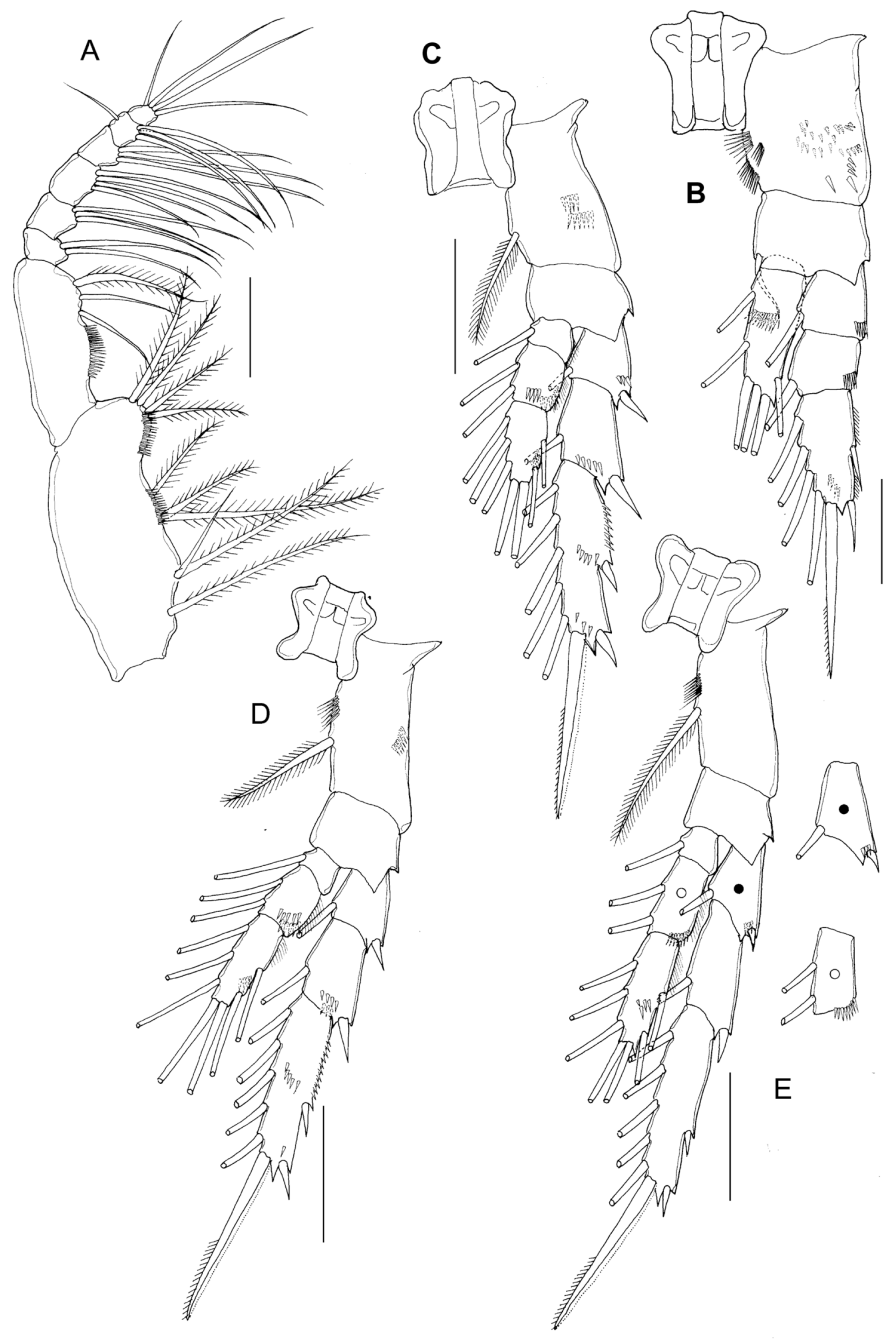
*Parvocalanus leei* sp. n., paratype adult female. **A** maxilliped **B** leg 1, dorsal view **C** leg 2, dorsal view **D** leg 3, dorsal view **E** leg 4, dorsal view. All scale bars 0.05 mm.

**Figure 5.**
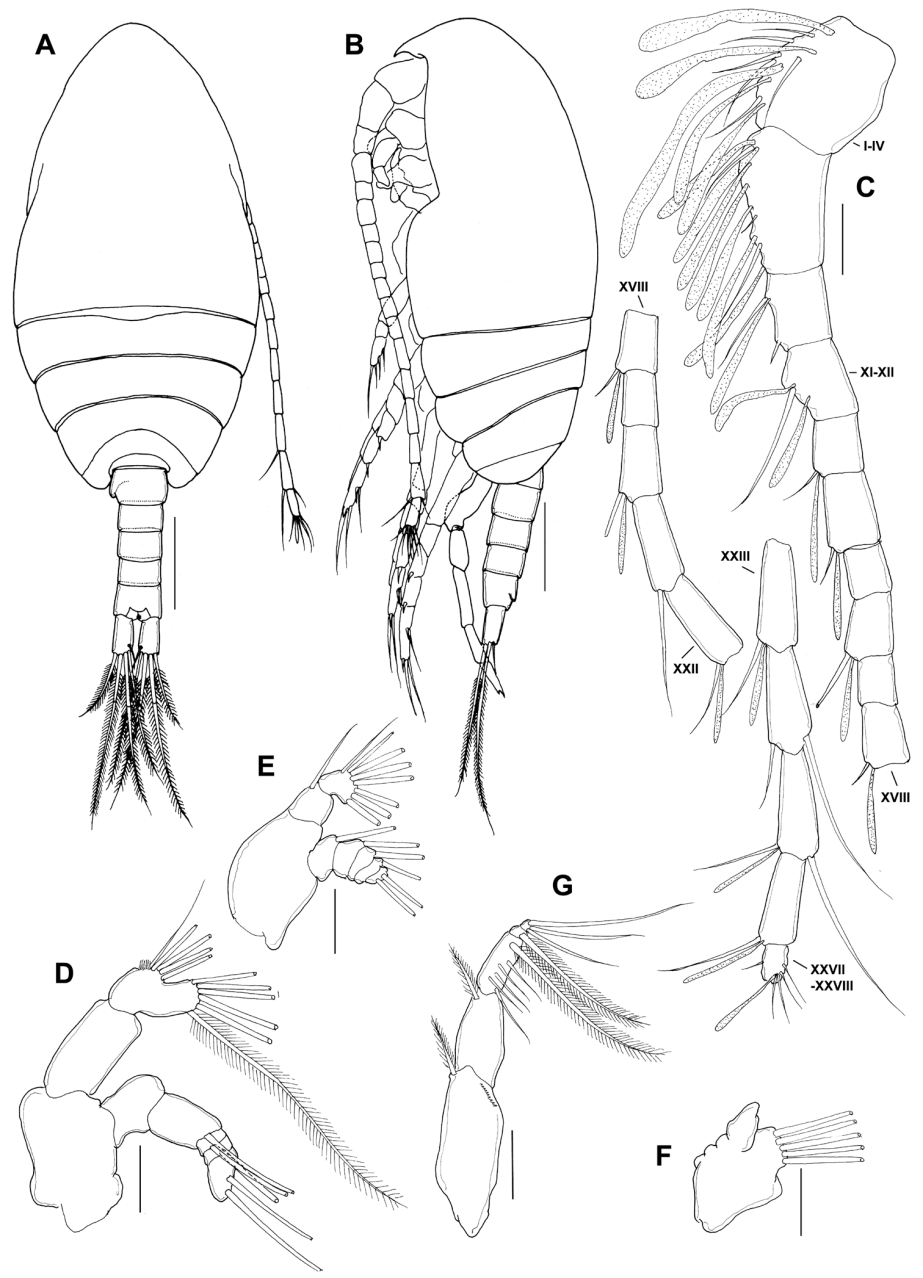
*Parvocalanus leei* sp. n., paratype adult male. **A** habitus, dorsal view **B** habitus, lateral view **C** antennule **D** antenna **E** mandible **F** maxillule **G** maxilliped. Scale bars: **A, B** = 0.1 mm; **C–G** = 0.025 mm.

**Figure 6.**
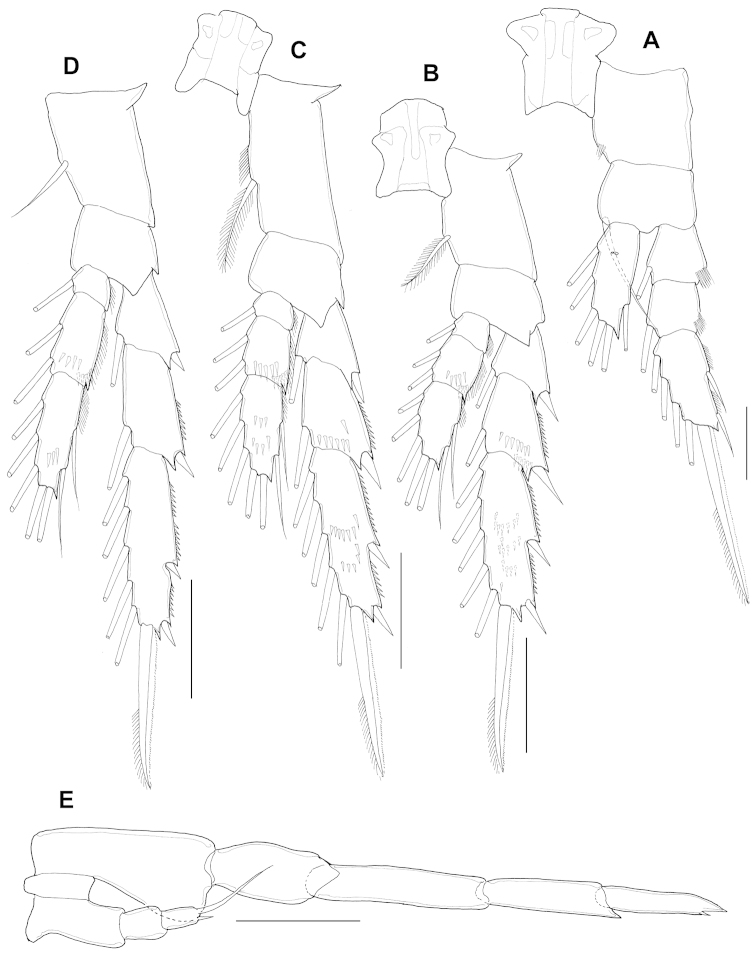
*Parvocalanus leei* sp. n., paratype adult male. **A** Leg 1, dorsal view **B** Leg 2, dorsal view **C** Leg 3, dorsal view **D** Leg 4, dorsal view **E** Leg 5, dorsal view. All scale bars 0.05 mm.

Figs 2 , 3 , 4 , 5 , 6

##### Type material.

Adult female holotype, 0.93 mm (NIBRIV0000302101) and adult male allotype, 0.62 mm (NIBRIV0000302102) preserved undissected in 70% ethanol, collected from the Yellow Sea, Korea (34°46'10"N, 126°20'24"E). Paratypes: 20 females (NIBRIV302103) and 10 males (NIBRIV302104) preserved in 70% ethanol, 21 August 2013. Dissected paratypes (5 females, 3 males) are kept in collection of the senior author. Description below is based on paratypes.

##### Type locality.

Shallow waters of Mokpo (34°46'10"N, 126°20'24"E), Western Korea.

##### Etymology.

The species is named after Mrs. Jungah Lee, wife of senior author (S.Y. Moon), as a small token of appreciation for her encouragement and support to senior author.

##### Description.

**Female.** (Based on female paratype). Body (Fig. 2A, B) 0.92 mm, plump. Prosome length 2.7 times as long as urosome including caudal rami, 3.6 times as long as urosome excluding caudal rami. Prosome 5-segmented: cephalosome and first pedigerous somite completely fused, 1.49 times longer (467 µm) than wide (313 µm); fourth and fifth pedigerous somites completely separated (Fig. 2A, B). Proportional length (%) of prosomites as 68.2:11.6:10.4:5.5:4.3=100. Rostrum (Fig. 3A) short, broad, about 23 µm long. Urosome 4-segmented (Figs 2A, B, 3B): genital double-somite symmetrical, swollen anterolaterally, 1.12 times wider (81 µm) than long (72 µm); genital system remarkably symmetrical with paired gonopores located each side, genital operculum (Fig. 3C) located midventrally, rounded, about one-third as long as genital double-somite. Caudal rami (Figs 2A, B, 3B) nearly symmetrical, 2.4 times longer (66 µm) than wide (27 µm), each with row of hairs on anterior inner margin and 5 caudal setae: seta II and VI spiniform; III, IV, and VII setiform and plumose. Proportional length (%) of urosomites and caudal rami as 28.3:9.5:10.4: 27.2: 24.6 = 100.

Antennule 25-segmented (Fig. 2C); extending to midlength of anal somite; ancestral segments II to IV and XXVII-XXVIII completely fused. Segmentation and setation pattern as follows: I-2s + 1ae, II-IV – 4s + 1ae, V – 1s + 1ae, VI – 1s, VII – 1s + 1ae, VIII – 1s, IX – 1s + 1ae, X – 1 spine + 1s, XI – 1s + 1ae, XII – 1s + 1ae, XIII – 1s, XIV – 1 spine+ 1ae, XV – 1s, XVI – 1s + 1ae, XVII – 1s, XVIII – 1s + 1ae, XIX – 1s + 1ae, XX – 1s, XXI – 1s + 1ae, XXII – 1s + 1ae, XXIII – 1s, XXIV – 1s + 1s, XXV – 1s + 1s, XXVI – 1s + 1s, XXVII-XXVIII – 5s + 1ae. Ancestral segments I to XXV with row of spinules on posterior surface. Ancestral segment X and XIV with short spiniform process on distal margin of dorsal surface.

Antenna (Fig. 3D) biramous; coxa with two setae; basis with single seta; endopod 2-segmented, first endopodal segment with 2 setae; second endopodal segment with 8 setae about midway of inner margin, 7 setae terminally, and oblique row of tiny spinules midway and subdistally on outer margin; exopod 7-segmented, setal formula 1, 3, 1, 1, 1, 1, 4.

Mandible (Fig. 3E, F): gnathobase well developed, cutting edge with short teeth and dorsal single seta (Fig. 3F). Mandibular palp biramous; basis with 4 setae; exopod 5-segmented, setal formula 1, 1, 1, 1, 2; endopod 2-segmented, proximal and distal segments with 4 and 11 setae, respectively; oblique row of tiny spinules subterminally on distal segment.

Maxillule (Fig. 3G): praecoxa and coxa partially fused; praecoxal arthrite with 14 elements, and with several rows of spinules on anterior surface; coxal endite with 3 setae, coxal epipodite with 9 setae; proximal basal endite with 3 setae, distal basal endite with 4 setae; endopod 3-segmented, setal formula 3, 3, 7; exopod unsegmented with 11 marginal setae.

Maxilla (Fig. 3H): precoxa and coxa completely fused, each with two endites, posteromedial surface furnished with setules; proximal praecoxal endite with 6 setae, distal endite with 3 setae; coxal endites each with 3 setae; coxal epipodite seta present; basis with 4 setae and row of spinules subterminally; endopod 4-segmented, first and second segments incompletely separated with setal formula of 1, 2, 2, 3.

Maxilliped (Fig. 4A): syncoxa robust with setal formula 1, 2, 3, 4 and oblique rows of spinules on anterior surface; basis with 3 setae and setules on medial surface; endopod 6-segmented, first and second segments completely separated with setal formula 2, 3, 4, 3, 3+1, 4.

P1 (Fig. 4B): coxa with spinules anterolaterally and subterminally; basis with inner seta; exopod 3-segmented, first to third exopodal segments with spinules subterminally and terminally; endopod unsegmented, with row of spinules anteriomedially.

P2 (Fig. 4C): coxa with spinules on posterior magin; basis unadorned; exopod 3-segmented, first and second segments with row of spinules on anterodistally, third exopodal segment with denticles on outer proximal edge; endopod 3-segmented, first segment smooth; second segment with spinules anterodorsally and posterodistally; third endopodal segment with row of spinules posterolaterally.

P3 (Fig. 4D): coxa with spinules posteromedially; basis unadorned; exopod 3-segmented, first segment smooth; second segment with row of spinules anterodistally and posterodistally; third exopodal segment with denticles on outer proximal edge and spinules on anterior margin; endopod 3-segmented, first segment smooth; second segment with spinules anterodorsally and posterodistally; third endopodal segment with row of spinules anterolaterally.

P4 (Fig. 4E): basis unadorned; exopod 3-segmented, first segment with row of spinules posterodistally; spinules absent on anterior margin of third exopodal segment; endopod 3-segmented, first segment smooth; second segment with spinules posterodistally; third endopodal segment with row of spinules anteromedially.

Armature formula of swimming legs 1–4 (P1–P4) as follows (Roman numerals indicate spines, Arabic numerals indicate setae):

**Armature formula of swimming legs 1–4 (P1–P4):** 

Legs	Coxa	Basis	Exopod segment	Endopod segment
P1	0-0	0-1	0-1;0-1;II,I,4	1,2,3
P2	0-1	0-0	I-1;I-1;II,I,5	0-1;0-2;2,2,3
P3	0-1	0-0	I-1;I-1;II,I,5	0-1;0-2;2,2,3
P4	0-1	0-0	I-1;I-1;II,I,5	0-1;0-2;2,2,3

P5 (Fig. 2D) 2-segmented, proximal segment smooth, unarmed; distal segment 2.15 times as long (31 µm) as wide (14 µm) with row of spinules subdistally and with two unequal terminal spines, inner distal spine longest, denticulated along distal part of outer margin.

**Male.** (Based on male paratype): Body (Fig. 5A, B) 0.53 mm, plumper than female. Prosome length 2.6 times as long as urosome including caudal rami. Prosome 5-segmented: cephalosome without dorsal hump and first pedigerous somite completely fused, 1.31 times longer (281 µm) than wide (213 µm); fourth and fifth pedigerous somites completely separated (Fig. 5A, B). Proportional length (%) of prosomites 60.5:13.5:13.5:12.5=100. Rostrum as in female. Urosome 5-segmented; first urosomal somite longest; proportional length (%) of urosomites 25.2: 20.3: 16.6: 14.6: 23.3=100. Caudal rami nearly symmetrical, about 2.2 times longer than wide, each with 5 setae, setae I and II wanting.

Antennule (Fig. 5C) 19-segmented, extending to distal part of third urosomite; ancestral segments I-IV, V-VIII, IX-X, XI-XII, and XXVII-XXVIII completely fused. Segmentation and setation as follows: segment 1 (fused ancestral segments I-IV), 7s+5ae; segment 2 (fused V-VIII), 3s+7ae; segment 3 (fused IX-X), 1s+1 spine+1ae; segment 4 (fused XI-XII), 1s+ 2ae; segments 5 (XIII) and 6 (XIV), 1s+1ae each; segment 7 (XV), naked; segment 8 (XVI), 1s+1ae; segment 9 (XVII), naked; segment 10 (XVIII), 1s+1ae; segment 11 (XIX), naked; segment 12 (XX), 1s+1ae; segment 13 (XXI), 1s; segment 14 (XXII), 1s+1ae; segment 15 (XXIII), 1s+1ae; segment 16(XXIV)1s+1s; segment 17 (XXV), 1s+1s+1ae; segment 18 (XXVI), 1s+1ae; segment 19 (XXVII-XXVIII), 5s+1ae.

Antenna (Fig. 5D) biramous but vestigial; coxa and basis completely fused, both unarmed; endopod 2-segmented, proximal endopodal segment naked; distal segment with 5 setae about midway of inner margin and with 6 terminal setae; exopod 5-segmented, setal formula 0, 1, 1, 1, 2.

Mandible (Fig. 5E) coxal gnathobase lacking; basis unarmed; exopod 5-segmented, setal formula 1, 1, 1, 1, 2; endopod 2-segmented, first endopodal segment with single seta, second endopodal segment with 8 setae.

Maxillule (Fig. 5F) vestigial presumed coxal epipodite with 5 setae.

Maxilla (not figured) vestigial.

Maxilliped (Fig. 5G): comprising robust syncoxa, basis, and 3-segmented endopod; syncoxa with a single seta and row of tiny spinules on inner distal edge; basis medially with single stout seta; proximal endopodal segment with 6 setae, of which distal seta robust; second segment with single seta; distal segment with 3 setae.

Swimming legs seta and spine formula and ornamentation (Fig. 6A–D) generally as in female, but with some differences, as follows: P1 (Fig. 6A) lacks posterior spinules on coxa, the basis and endopod are unadorned, and the third exopodal segment lacks of row of spinules on posterior surface; P2 (Fig. 6B) has the second and distal endopodal segments with denticles on outer edge; distal endopodal segment without row of spinules on mediolateral margin; P3 (Fig. 6C) has the second and distal endopodal segments with denticles on outer edge; second exopodal segment without row of spinules on the posterodistal margin; and P4 (Fig. 6D) has the second and distal endopodal segments with denticles on outer edge; and first exopodal segment without row of spinules on the anterodistal margin.

P5 (Fig. 6E) strongly asymmetrical and uniramous: right P5 5-segmented and longer than second urosomal segment; basis and first exopodal segment unarmed; second exopodal segment with pointed process on distomedial angle; distal segment with two pointed processes, inner tiny. Left leg 3-segmented; distal segment with tiny outer apical spine, inner apical spine long, 9 times as long as outer spine.

##### Variation.

Body length ranged from 0.75–0.92 mm (mean±sd, 0.84±0.05, *N*=10) in females and 0.49–0.69 mm (mean±sd, 0.55±0.07, *N*=6) in males. Variability was found in number of spinules on posterior surface of P1–P4 in both sexes, on posterodistal margin of female P5, on the length/width ratio of second segment of female P5 (2.15–2.54 times as long as wide; mean±sd; 2.31±0.12, *N*=5), and on ornamentation of denticles on the second and distal exopodal segments of P2–P4 in female.

##### Distribution.

*Parvocalanus leei* sp. n. generally occurred together with other paracalanids, such as *Bestiolina coreana* Moon, Lee & Soh, 2010, *Parvocalanus crassirostris*, and *Paracalanus parvus* s. l. at the collection sites in the Yellow Sea, Korea on 21 August 2013. This new species is predominantly found in shallow waters with temperature above approximately 20 °C and 32 psu in the Mokpo Harbor, Western Korea.

##### Remarks.

The adult female of *Parvocalanus leei* sp. n. is very similar to *Parvocalanus arabiensis* (Kesarkar & Anil, 2010), *Parvocalanus crassirostris*, *Parvocalanus latus* Andronov, 1972, and *Parvocalanus scotti* (Früchtl, 1923). All them share the short and blunt rostrum and the elongate distal segment of P5, with the inner terminal spine less than three times the length of the outer terminal spine. Nevertheless, the new species differs from *Parvocalanus arabiensis* as follows: (1) the body length is higher than 0.7 mm in the new species, but less than 0.7 mm in *Parvocalanus arabiensis*; (2) the antennule extends up to the medial margin of third urosomite in the new species, but only to the posterior margin of genital double-somite in *Parvocalanus arabiensis*; (3) the endopod of P1 is unsegmented in the new species, but 2-segmented in *Parvocalanus arabiensis*; (4) the inner spine of P5 is less than 1.7 times longer than outer terminal spine of P5 in the new species, but more than 1.7 times longer than in *Parvocalanus arabiensis*; and (5) there is no ornamentation of denticles on the distal edge of the third exopodal segment of P4 in the new species, vs. denticles present in *Parvocalanus arabiensis*.

The female of *Parvocalanus leei* closely resembles *Parvocalanus crassirostris*, but is larger (more than 0.7 mm in length compared to less than 0.7 mm); the fourth and fifth pedigerous somites are separated (vs. partially fused in *Parvocalanus crassirostris*); the antennules extend to the medial margin of anal somite (vs. approximately to second urosomite in *Parvocalanus crassirostris*); the length/width ratio of the distal segment of P5 is lower than 2.5 (vs. more than 3 in *Parvocalanus crassirostris*); and there is a row of spinules on the distal end of the second segment of P5 (vs. row absent in *Parvocalanus crassirostris*).

The new species shares with *Parvocalanus latus* the similar body shape and the P5 ornamentation in the female, but differs in the following features: (1) the body is more than 0.7 mm in length (vs. less than 0.7 mm in *Parvocalanus latus*); (2) the antennule is comparatively shorter, reaching only the medial margin of anal somite (vs. reaching the end of caudal rami in *Parvocalanus latus*); and (3) the genital double-somite is swollen anterolaterally in the new species (vs. somite not swollen in *Parvocalanus latus*).

The female of *Parvocalanus leei* can be readily differentiated from *Parvocalanus scotti* based on the following features: (1) the body is more than 0.7 mm (vs. less than 0.7 mm in *Parvocalanus scotti*); (2) the antennule extends only to the medial margin of anal somite (vs. to the distal margin of caudal rami in *Parvocalanus scotti*); (3) the length/width ratio of caudal rami is higher than 2 in the new species (vs. less than 2 in *Parvocalanus scotti*), and (4) the length/width ratio of second segment of P5 is less than 3 (vs. more than 3 times in *Parvocalanus scotti*).

## Discussion

Kesarkar and Anil (2010) distinguished between *Paracalanus* Boeck, 1864 and *Parvocalanus* based on the following characteristics of the female, because all species had been previously described based only on the female: (1) basis of P1 with inner edge seta (vs. without inner edge seta in *Parvocalanus*); (2) P1 endopod 2-segmented (vs. endopod unsegmented or 2-segmented in *Parvocalanus*); and (3) second endopodal segment of P1 with 5 setae (vs. 6 setae in *Parvocalanus*). But these characteristics overlap in the two genera. Indeed, the genus *Parvocalanus* shows many similarities with *Paracalanus*, but differs in the following features: (1) rostrum short, broad and bifurcated in both sexes; (2) distal segment of P5 terminal spines short in the female; and (3) absence of a medial keel-like dorsal hump on the cephalosome of male. However, five species of *Parvocalanus* have been described and/or illustrated as having an inner seta on the basis of P1, namely: *Parvocalanus arabiensis*, *Parvocalanus dubia*, *Parvocalanus leei* sp. n., *Parvocalanus scotti*, and *Parvocalanus serratipes*. A molecular phylogeny recently published discriminated the genera *Parvocalanus* and *Paracalanus* with well-supported nodes, with *Parvocalanus* placed as sister to the rest of paracalanid genera (Cornils and Blanco-Bercial 2013). But the morphological phylogeny assessed in parallel by the same authors failed to separate the two genera (Cornils and Blanco-Bercial 2013), although it should be remarked that the morphological dataset used was extremely poor due to the poorness of the original species descriptions.

*Parvocalanus leei* is distinguished from the rest of members of its genus based on the following characteristics of the female: fourth and fifth pedigerous somites completely separated, large size (more than 0.7 mm), and presence of spinules on the distal end of distal segment of P5. These differences are shown in Table 1. In the present study, we have re-examined the following combination of female features in order to separate species: (1) body shape and size; (2) relative length of antennule; (3) fusion of fourth and fifth pedigerous somites; (4) presence/absence of spinules on second endopodal and exopodal segments of P2 and P4; (5) presence/absence of spinules on distal end of distal segment of P5; (6) length/width ratio of second segment of P5; and (7) length ratio between inner and outer terminal spines of P5.

**Table 1.** Comparison of morphological characteristics of female *Parvocalanus* spp. A1, antennules; P1, swimming leg 1; P2, swimming leg 2; P4, swimming leg 4; P5, fifth leg.

Character	Swimming leg (P)	*Parvocalanus arabiensis* (Kesarkar & Anil, 2010)	*Parvocalanus crassirostris* (F. Dahl, 1894)	*Parvocalanus dubia* (Sewell, 1912)	*Parvocalanus elegans* Andronov, 1972	*Parvocalanus latus* Andronov, 1972	*Parvocalanus leei* sp. n.	*Parvocalanus serratipes* (Sewell, 1912)	*Parvocalanus scotti* (Früchtl, 1923)
Body length (mm)		0.55–0.60	0.5	0.74	0.48–0.50	0.42–0.47	0.75–0.92	1.1	0.64–0.67
Body form		Broad and short	Broad and short	Broad and short	Narrow and long	Broad and short	Broad and short	Broad and short	Broad and short
Fourth and fifth pedigerous somites		Partially fused	Partially fused	Completely fused	Separated	Separated	Separated	Completely fused	Partially fused
A1 extending to:		Almost to end of genital double-somite	Almost to second urosomite	Midlength to first urosomite	Almost to end of anal somite	Beyond caudal rami	Midlength to anal somite	Midlength to first urosomite	Beyond caudal rami
Basis of P1 inner seta		Present	X	Present	Absent	Absent	Present	Present	Present
Endopod of P1		2-segmented	X	2-segmented	Unsegmented	Unsegmented	Unsegmented	2-segmented	Unsegmented
Number of spinules on dorsal surface of first to third exopodal segments of P1–P4	P2	5, 4, 6	X	3, 0, 4	Absent	Absent	3, 5, 8	0, 4, 5	0, 5, 5
	P3	Absent	X	X	0, 6, 0	0, 6, 0	0, 4, 6	0, 4, 5	0 7, 7
	P4	Absent	X	X	Absent	Absent	Absent	Absent	0, 7, 0
Number of spinules on dorsal surface of second endopodal segment of P2–P4	P2	0, 3, 0	X	0, 4, 0	0, 3, 0	0, 3, 0	0, 4, 0	0, 6, 0	0, 4, 0
	P3	0, 7. 0	X	X	0, 4, 2	0, 4, 2	0, 5, 0	0, 4, 0	0, 6, 3
	P4	Absent	X	X	0, 3, 2	0, 3, 2	0, 0, 4	Absent	0, 4, 3
Length/width ratio of distal segment of P5		Twice as long as wide	Three times as long as wide	Four times as long as wide	Three times as long as wide	Twice as long as wide	Twice as long as wide	Four times as long as wide	Three times as long as wide
Row of spinules on distal segment of P5		Present	Absent	Present	Present	Absent	Present	Present	Present
Length ratio between inner and outer terminal spines of P5		2	< 2	= 3	3	> 3	< 2	> 2	> 2

*Parvocalanus crassirostris* was originally described by F. Dahl (1894) as *Paracalanus crassirostris* from the mouth of the river Tocantins, Brazil, but the description was rather incomplete and based on the female only (see F. Dahl 1894: taf. I, figs 27 and 28). The populations of this species from estuarine and shallow waters of Korea closely resemble the Brazilian population, both having two short apical spines on the distal segment of P5, but the female of *Parvocalanus leei* is larger than *Parvocalanus crassirostris*, its fourth and fifth pedigerous somites are completely separated and the length/width ratio of distal segment of its P5 is lower (see Table 1). The populations of *Parvocalanus crassirostris* from Japanese waters are very similar to *Parvocalanus leei*, but differ in having an inner seta on the coxa of P1 in both sexes, a distal segment of female P5 devoid of a distal row of spinules, the male left P5 is 3-segmented (N=5) with the long apical spine (39 µm) 6.5 times as long as the short outer spine (6 µm), and the body is larger in both sexes (see Hiromi 1981). *Parvocalanus crassirostris* has a worldwide distribution throughout temperate and tropical regions (Razouls et al. 2005–2014) despite its morphological homogeneity. Kesarkar and Anil (2010) described the populations of this species from the Mondovi and Zuari estuaries, Goa, West coast of India, as a new species *Parvocalanus arabiensis* (Kesarkar and Anil 2010). However, these authors might have overlooked some previous morphological studies of *Parvocalanus crassirostris* (Tanaka 1960; Shen and Lee 1963; Hiromi 1981; Bradford-Grieve 1994). Additionally, most of the *Parvocalanus* species were not described following modern standards and most of them need to be redescribed. Thus, the taxonomy, morphological variability and distribution of *Parvocalanus crassirostris* is not well understood. These facts suggest that a more detailed research on its geographical variation in terms of morphological and molecular features is necessary for a better understanding of its evolutionary history.

## Taxonomic review on *Paracalanus arabiensis* Kesarkar & Anil, 2010

*Paracalanus arabiensis* was originally described by Kesarkar and Anil (2010) based on 11 adult females collected from Mondovi and Zuari estuaries, Goa, west coast of India. The assignment of this taxon to *Paracalanus* was based on the examination of the literature, where figures of some of the presumed diagnostic features of the genus, such as presence of inner edge seta on basis of female P1, P1 endopod 2-segmented, and second endopodal segment of P1 with 5 setae were shown. However, it shares the generic characteristics of *Parvocalanus* (see Andronov 1970; Hiromi 1987; Boxshall and Halsey 2004). Two major differences between *Parvocalanus arabiensis* and the members of *Parvocalanus* are the presence of inner seta on the basis of P1, and of a 2-segmented endopod in P1 with 5 setae on the distal segment in the former species. Since the presence or absence of inner seta on the P1 basis has been historically used to define some species of *Parvocalanus*, we believe this feature is not relevant enough as to put this Arabian taxon in a genus different to *Parvocalanus*. We consider more appropriate to slightly modify the generic diagnosis of *Parvocalanus* to include “basis of P1 with or without inner seta” and “endopod of P1 unsegmented or 2-segmented” to accommodate *Parvocalanus arabiensis* within this genus. *Parvocalanus arabiensis* (Kesarkar & Anil, 2010), comb. n. resembles *Parvocalanus crassirostris* in the small body size, the short and bifurcate rostrum, ending in two acute points, and in the presence of two short terminal spines on the female P5. But they can be readily distinguished based on rostrum appearance; the relative length of terminal spines of female P5; and the presence/absence of a medial keel-like dorsal hump on the cephalosome of male.

As an update we report that *Parvocalanus* has eight nominal species including the one described herein: *Parvocalanus arabiensis*, *Parvocalanus crassirostris*, *Parvocalanus dubia* (Sewell, 1912), *Parvocalanus elegans*, *Parvocalanus latus* Andronov, 1972, *Parvocalanus leei* sp. n., *Parvocalanus scotti*, and *Parvocalanus serratipes* (Sewell, 1912). A key to all genera and species of Paracalanidae is provided below.

### Key to the genera of Paracalanidae (amended from Boxshall and Halsey 2004)

**Table:** 

1	Distal endopodal segment of P2 setal formula 1, 2, 2	***Mecynocera***
–	Distal endopodal segment of P2 setal formula not 1, 2, 2	**2**
2	Distal endopodal segment of P2 setal formula 1, 2, 3; female P5 reduced	**3**
–	Distal endopodal segment of P2 setal formula 2, 2, 3; female P5 not reduced	**4**
3	Outer margins of second and distal exopodal segments of P2 to P4 ornamented with strong spinules; distal endopodal segment of P3 and P4 with setal formula 2, 2, 3; female P5 strongly reduced	***Acrocalanus***
–	Outer margins of second and distal exopodal segments of P2 to P4 lacking spinular ornamentation; distal endopodal segment of P3 and P4 with setal formula 1, 2, 3; female P5 strongly reduced to pair of rounded lobes	***Bestiolina***
4	Right fifth leg lacking in both sexes; outer margins of third exopodal segment of P2 to P4 lacking spinulations in female	***Delibus***
–	Fifth legs symmetrical in female; small right P5 present in male	**5**
5	Inner seta on basis of P1 present; outer distal margin of third exopodal segment of P2 to P4 conspicuously serrated	***Paracalanus***
–	Inner seta on basis of P1 absent or present; outer distal edges of third exopodal segment of P2 to P4 smooth in female	**6**
6	Median keel-like dorsal hump present on the cephalosome of male; rostrum with slender paired filaments in both sexes; male right P5 3 or 4-segmented; female P5 3 or 4-segmented	***Calocalanus***
–	Medial keel-like dorsal hump absent on the cephalosome of male; rostrum short and broad, bifurcate, terminating in two acute points; male right P5 4-segmented; female P5 endopod 1 or 2-segmented	***Parvocalanus***

### Key to the species of *Parvocalanus* (based on adult female)

**Table:** 

1	Fourth and fifth pedigerous somites completely fused; distal segment of female P5 long and slender, approximately 4 times as long as wide, with row of spinules on distal end	**2**
–	Fourth and fifth pedigerous somites not fused; distal segment of female P5 less than 5 times as long as wide, with/without row of spinules on distal end	**3**
2	Body length less than 1 mm; dorsal surface of second endopodal segment of P2 without spinulation	***Parvocalanus dubia* (Sewell, 1912)**
–	Body length more than 1 mm; dorsal surface of second endopodal segment of P2 with spinulation	***Parvocalanus serratipes* (Sewell, 1912)**
3	Body narrow and long; fourth and fifth pedigerous somites completely separated	***Parvocalanus elegans* Andronov, 1972**
–	Body broader and shorter; fourth and fifth pedigerous somites completely separated or partially fused	**4**
4	P 1endopod 2-segmented; Al extending almost to end of genital double-somite	***Parvocalanus arabiensis* (Kesarkar & Anil, 2010)**
–	P1 endopod unsegmented; A1 extending over to genital double-somite	**5**
5	Inner seta on basis of P1 absent; A1 extending beyond caudal rami; inner terminal spine more than three times length of outer terminal spine	***Parvocalanus latus* Andronov, 1972**
–	Inner seta on basis of P1 absent or present; A1 not reaching caudal rami; inner terminal spine less than three times length of outer terminal spine	**6**
6	Inner seta on basis of P1 absent; fourth and fifth pedigerous somites partially fused; A1 extending approximately to second urosomite; row of spinules on distal segment of P5 absent	***Parvocalanus crassirostris* (F. Dahl, 1894)**
–	Inner seta on basis of P1 present; fourth and fifth pedigerous somites partially fused or completely separated; A1 extending over to second urosomite; row of spinules on distal segment of P5 present	**7**
7	Fourth and fifth pedigerous somites completely separated; A1 extending to medial margin of anal somite; dorsal surface of second exopodal segment of P4 without spinulation; length/width ratio of distal segment of P5 lower than 3	***Parvocalanus leei* sp. n.**
–	Fourth and fifth pedigerous simites partially fused; A1 extending to beyond caudal rami; dorsal surface of second exopodal segment of P4 with spinules; length/width ratio of distal segment of P5 higher than 3	***Parvocalanus scotti* (Früchtl, 1923)**

## Supplementary Material

XML Treatment for Parvocalanus leei
